# Effects of cardiovascular exercise early after stroke: systematic review and meta-analysis

**DOI:** 10.1186/1471-2377-12-45

**Published:** 2012-06-22

**Authors:** Oliver Stoller, Eling D de Bruin, Ruud H Knols, Kenneth J Hunt

**Affiliations:** 1 Institute for Rehabilitation and Performance Technology, Bern University of Applied Sciences, Burgdorf, Switzerland; 2 Department of Epidemiology, Maastricht University and Caphri Research School, Maastricht, Netherlands; 3 Institute of Human Movement Sciences and Sport, ETH Zurich, Zurich, Switzerland; 4 Physiotherapy Occupational Therapy Research, Center for Clinical Research, University Hospital Zurich, Zurich, Switzerland

## Abstract

**Background:**

Previous studies have shown the beneficial effects of aerobic exercise in chronic stroke. Most motor and functional recovery occurs in the first months after stroke. Improving cardiovascular capacity may have potential to precipitate recovery during early stroke rehabilitation. Currently, little is known about the effects of early cardiovascular exercise in stroke survivors. The aim of this systematic review was to evaluate the effectiveness of cardiovascular exercise early after stroke.

**Methods:**

A systematic literature search was performed. For this review, randomized and non-randomized prospective controlled cohort studies using a cardiovascular, cardiopulmonary or aerobic training intervention starting within 6 months post stroke were considered. The PEDro scale was used to detect risk of bias in individual studies. Inter-rater agreement was calculated (kappa). Meta-analysis was performed using a random-effects model.

**Results:**

A total of 11 trials were identified for inclusion. Inter-rater agreement was considered to be “very good” (Kappa: 0.81, Standard Error: 0.06, CI95%: 0.70–0.92), and the methodological quality was “good” (7 studies) to “fair” (4 studies). Peak oxygen uptake data were available for 155 participants. Pooled analysis yielded homogenous effects favouring the intervention group (standardised mean difference (SMD) = 0.83, CI95% = 0.50–1.16, Z = 4.93, P < 0.01). Walking endurance assessed with the 6 Minute Walk Test comprised 278 participants. Pooled analysis revealed homogenous effects favouring the cardiovascular training intervention group (SMD = 0.69, CI95% = 0.45–0.94, Z = 5.58, P < 0.01). Gait speed, measured in 243 participants, did not show significant results (SMD = 0.51, CI95% = −0.25–1.26, Z = 1.31, P = 0.19) in favour of early cardiovascular exercise.

**Conclusion:**

This meta-analysis shows that stroke survivors may benefit from cardiovascular exercise during sub-acute stages to improve peak oxygen uptake and walking distance. Thus, cardiovascular exercise should be considered in sub-acute stroke rehabilitation. However, concepts to influence and evaluate aerobic capacity in severely affected individuals with sub-acute stroke, as well as in the very early period after stroke, are lacking.

Further research is needed to develop appropriate methods for cardiovascular rehabilitation early after stroke and to evaluate long-term effects of cardiovascular exercise on aerobic capacity, physical functioning, and quality-of-life.

## Background

Each year 15 million people worldwide experience a stroke. About one third of this population will remain disabled [1]. According to European population projections from the United Nations the number of new cases of stroke will increase from 1.1 million to 1.5 million in 2025 [2]. Furthermore, it was estimated that stroke will occur in 35% of the population over the age of 65, a group that will increase in proportion due to demographic shifts in most populations [3]. This will presumably lead to an increased need for rehabilitation programmes to enhance recovery, improve functional status and quality-of-life, thus leading to future challenges for societies, and health-care and financial systems. Rehabilitation strategies for stroke survivors have focused primarily on restoring reduced motor control of the affected side as well as postural control [4,5]. However, less than a third of the variance in disability after stroke can be explained by the extent of neurological impairment [6]. Around 75% of post-stroke individuals exhibit cardiac disease [7,8]. Thus stroke survivors may be more disabled by associated cardiac disease than by the stroke incident itself [9].

Individuals after stroke generally have low endurance for exercise as a secondary consequence of immobility [10,11]. Previous work has shown that maximum oxygen uptake (VO_2_max) is reduced to 10–17 ml/kg/min within 0–30 days after the stroke [12-14], and does not rise above 20 ml/kg/min after six months [11,14-16]. These values are 25–45% lower than VO_2_max in age-matched, healthy subjects [17,18]. This early and persistent decline in aerobic capacity can delay or inhibit participation in a therapeutic exercise programme, complicate the rehabilitation process and long-term post-stroke course of care, and limit the ability of the individual to perform functional activities independently [8,19]. Previous work has shown that subjects with chronic stroke will reach their maximum aerobic capacity during ADL activities [18]. Therefore, a minor increase in aerobic capacity could mean the difference between dependency and independence during all daily living activities.

From these observations it can be concluded that cardiovascular training early after stroke may be a useful treatment option. Several controlled clinical trials evaluated the effect of cardiovascular exercise in individuals post-stroke [11,20-26]. The results of these studies are summarised in a meta-analysis demonstrating significant homogenous standard effects sizes for improving aerobic capacity, walking speed, and walking endurance in individuals with stroke. However, the main focus of the studies included was on the chronic stage after stroke (>6 months after the initial event) [27]. Whether these findings can be generalised, and whether such training therefore is also feasible for a population in the acute and sub-acute stages following stroke, remains unclear.

Until now, little is known about the effects of cardiovascular training in the early stages of stroke. Given the fact that most motor and functional recovery occurs in the first months after stroke [28], there could be a hitherto neglected potential to precipitate motor recovery by improving cardiovascular capacity in individuals early after stroke. A physical exercise programme with an adequate emphasis on cardiovascular parameters might be hypothesised to induce a training effect, which could facilitate improved physical functioning and lead to enhanced quality-of-life.

The purpose of this systematic review is to provide an overview of the currently available evidence for the use of cardiovascular training in the early stages after stroke. The aim is to identify strategies that have the potential to affect physical functioning and that might be used in future early intervention type studies for individuals with stroke. The following specific questions were evaluated: (1) What is the level of evidence for cardiovascular exercise interventions to influence aerobic capacity and physical functioning implemented within six months after the initial stroke event?; (2) How soon after the initial stroke event is cardiovascular exercise introduced?; (3) What is the common practice for measurement of aerobic capacity early after stroke?

## Methods

### Protocol

Analysis methods and inclusion criteria were specified in advance and documented in the review protocol, which can be found in Additional file 1.

### Data sources and search strategy

We searched the databases MEDLINE/Premedline (OvidSP), EMBASE, Cochrane Library, CINAHL, and ISI Web of Science (WOS), and performed an additional focussed search where “stroke” had to be in the title or in the subject headings. The final search was done on 24 November 2011. We used a combination of medical subject headings (MeSH) and textwords as search terms, including the following main terms for the population: Stroke, Cerebral Stroke, Vascular Accident, Brain Vascular Accident, Apoplexy, Cerebrovascular Apoplexy, Cerebrovascular Stroke, CVA (Cerebrovascular Accident), Cerebrovascular Accident, Acute Stroke, Acute Cerebrovascular Accident, Acute = 0–6 months post stroke, age >18 years. For the intervention of interest: cardiovascular training, cardiopulmonary training, cardiorespiratory training, aerobic training, endurance training, exercise, endurance exercise, ergometry, cycling, rowing, treadmill. For the outcomes of interest: cardiovascular fitness, aerobic fitness, condition, endurance, physical conditioning, VO_2_ maximal, VO_2_ maximum, VO_2_ peak, maximal oxygen uptake, heart rate, neural recovery, neural rehabilitation, functional recovery, function recovery, quality of life. Articles found through hand search by scanning reference lists of identified studies supplemented the search results.

### Eligibility criteria

We included randomized and non-randomized prospective controlled cohort studies considering cardiovascular training in the sub-acute stages after stroke. No language or publication date restrictions were imposed and only peer-reviewed journal articles were included. Participants (age >18 years) with initial stroke in the acute phase deemed medically stable enough to participate in an aerobic exercise intervention were considered. For the purpose of this systematic review, the phase after stroke was defined by the start of the intervention, whereas we defined “acute” as the first week after the stroke event, and “sub-acute” as 7 days – 6 month after stroke onset. All types of stroke and all severity levels were included. Furthermore, we considered only studies using cardiovascular, cardiopulmonary or aerobic training interventions. Typically, cardiovascular training is performed for extended periods of time on devices that allow for recruitment of large muscle groups or on ergometers (e.g. treadmill, cycling, rowing), or by utilising modes of activity such as walking, cycling, rowing or stair climbing. Any outcome representing an objective and/or subjective measure in the field of cardiovascular fitness, physical condition, endurance, oxygen uptake, heart rate, neural recovery, functional recovery, quality-of-life, etc. was included in this review. The primary outcome measures were directly related to physical functioning and/or physical performance using validated parameters or scales.

### Study selection

After duplicate citations were removed, two reviewers (OS, EDB) performed the identification and the eligibility assessment independently in a blinded standardized manner by scanning the titles, abstracts and keywords. Disagreements between reviewers were resolved by consensus. If no consensus was found, a third reviewer (KH) made the final decision.

### Data collection process

We developed a data extraction sheet based on the Cochrane Handbook for Systematic Reviews of Interventions data extraction template (The Cochrane Collaboration, Oxford, England). One reviewer (OS) extracted the data from included studies and a second reviewer (EDB) crosschecked the extracted data for accuracy. Disagreements were resolved by discussion between two reviewers (OS, EDB); if no agreement could be reached, a third author (KH) verified the data and made the final decision. We contacted authors by email or telephone for further information about unpublished and unclear data. We extracted the following information: study ID (author and year), title, design, intervention length, setting, number of participants, participants missing, inclusion criteria, exclusion criteria, age, sex, country, stroke type, location, severity, initial diagnostics, time since stroke, number of participants, experimental and control interventions, and additional outcome measures.

### Risk of bias in individual studies

Two independent reviewers (OS & EDB) assessed all studies for risk of bias. The PEDro scale was used for this assessment. The PEDro scale was developed to assess the methodological quality of physical therapy RCTs and is composed of 11 items rating internal validity (10 items) and external validity (1 item) of a clinical trial. The last item does not influence the internal or statistical validity of the trial and, in general, is not used to calculate the PEDro scale (partitioned) score. The reliability of the PEDro scale has been demonstrated [29]. Consistent with PEDro, we only considered the internal validity (10 items) to assess risk of bias, and used the following cut-points: 9–10 (excellent); 6–8 (good); 4–5 (fair); <4 (poor). Disagreements during the quality assessments were resolved by discussion between two review authors (OS & EDB); if no agreement could be reached, a third author (KH) verified the data and made the final decision.

### Risk of bias across studies

We assessed the possibility of publication bias by evaluating funnel plots of the trials’ mean differences for asymmetry, which can result from non-publication of small trials with negative results. Heterogeneity of effect sizes was evaluated by I^2^ statistics, where at least 50% was taken as an indicator of substantial heterogeneity.

### Analysis

Review Manager (The Cochrane Collaboration, Oxford, England) and Microsoft Excel (2011) were used to calculate mean differences (MD), standard deviation (SD), effects sizes (ES), confidence intervals (CI), and p-values (p). In a few instances, missing SDs of MDs and standard errors (SE) were calculated according to the Cochrane Handbook for Systematic Reviews (The Cochrane Collaboration, Oxford, England). For outcomes where sufficient data was available, we performed the meta-analyses by computing standardised mean differences (SMD) using random-effects models. Percentage agreement and Cohen’s kappa were calculated and interpreted in accordance with Landis and Koch’s benchmarks for assessing the agreement between raters: poor (≤0), slight (0.0 to 0.20), fair (0.21 to 0.40), moderate (0.41 to 0.60), substantial (0.61 to 0.80), and almost perfect (0.81 to 1.0) [30]. The PRISMA-statement was followed for reporting items of this systematic review [31].

## Results

### Study selection

**Figure 1:**
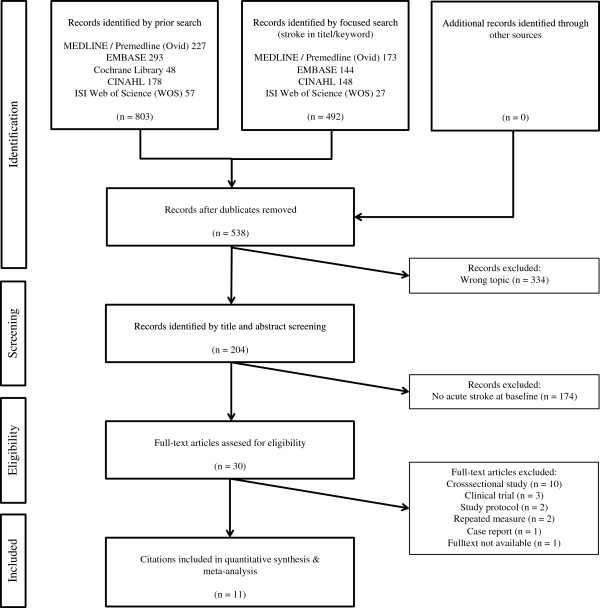
**Results of the systematic review.** Studies’ flow chart for the systematic review and meta-analysis.

The search of MEDLINE/Premedline (OvidSP), EMBASE, Cochrane Library, CINHAL, and ISI Web of Science (WOS) provided a total of 803 citations, and 492 citations with stroke in the title and/or keywords. 538 remained after adjusting for duplicates. 334 studies were discarded after reviewing the abstracts, because they clearly did not meet the inclusion criteria. 174 studies were excluded because subjects were not in the sub-acute phase after stroke at baseline of the interventions. The full text of the remaining 30 citations was examined in more detail. 19 of these studies did not meet the inclusion criteria. Finally, 11 studies fulfilled all criteria and were selected for review [32-42] (Figure 1).

### Study characteristics

The study characteristics are shown in Table 1. Ten studies were randomized controlled trials or randomized controlled pilot studies, and 1 study was a prospective controlled matched design [41]. All articles were published in peer-reviewed journals in English. The year of publication ranged from 1998 to 2011. All studies were carried out during inpatient rehabilitation and were from the United States (3), Germany (2), Israel (4), France (1), and Canada (1). The outcomes of the 3 studies from Katz-Leurer et al. [36-38] are based on the same sample, but used different outcome measures.

**Table 1:** Overview of included studies on cardiovascular exercise early after stroke

**Study ID**	**Participants**	**Inclusion criteria**	**Exclusion criteria**	**Exercise protocol**	**Additional interventions/control group**	**Outcomes (Instrument):**
						**+ = significant between-group difference**
						**0 = no difference between groups**
da Cunha_2002	15 participants	Gait deficit (gait speed <0.6 m/s), Functional Ambulation Category (FAC) 0–3, Mini-Mental State Exam >21, ability to stand and take steps, stable medical condition	Comorbidity, recent myocardial infarction or bypass surgery with complications (<4wk), uncontrolled health conditions, significant lower extremity degenerative joint disease, body weight >110 kg, history of bilateral cerebrovascular accident	Mode: 3 weeks of body weight supported treadmill training	Usual care/Usual care 3 h/day, conventional gait training	VO2consumption (5-minute walk): 0
	Age: 58.4			Intensity: Starting with 30% BWS, increasing progressively each session		VO2cost (5-minute walk): 0
	Days since stroke (intervention group): 15.7 ± 7.7 (9–28)			Duration: 20 min		Gait ability (FAC Scale): 0
	Moderately impaired, NIH: 4.5			Frequency: 5 x/week (15 sessions)		Gait speed (5MWT): 0
						Walking distance (5 minutes walk): 0
Duncan_1998	20 participants	30–90 days after stroke, FMMS 40–90, OPS 2–5.2, ambulatory with supervision and/or assistive device, living at home, living around 50 miles of the University of Kansas Medical Centre	Medical condition that interfered with outcome assessments or limited participation in submaximal exercise program, Mini-Mental State score <18, receptive aphasia that interfered with the ability to follow a 3-step command	Mode: 12 weeks of strength, balance and endurance training (leg cycle ergometry) in a home based setting	Usual care/Usual care, no endurance training	10MWT: + (*p* = 0.05 < 0.10)
	Age: 67.6			Intensity: Resistance progression after 2 sets of 10 repetitions		FM lower: + (*p* = 0.01 < 0.02)
	Days since stroke (intervention group): 66.0 ± N/A			Duration: 90 min		6MWT: 0
	Minor impaired, OPS: 2.7			Frequency: 3 x/week (36 sessions)		FMMS upper extremity: 0
						Barthel Index of ADL: 0
						Lawton Instrumental ADL: 0
						MOS36: 0
						Berg Balance Scale: 0
						Hand function (Jebsen Test): 0
Duncan_2003	100 participants	30–150 days after stroke, ability to ambulate 25 ft independently, FMMS 27–90, OPS 2–5.2, Mini-Mental State Score >16, ambulatory with supervision and/or assistive device, living at home, living around 50 miles of the University of Kansas Medical Centre	Serious cardiac conditions (hospitalisation for heart disease within 3 months, active angina serious cardiac arrhythmias, hypertrophic cardiomyopathy, severe aortic stenosis, pulmonary embolus, or infarction), oxygen dependence, severe weight-bearing pain, other serious organ system disease, life expectancy of <1 year	Mode: 12–14 weeks of strength, balance and endurance training (leg cycle ergometry) in a home based setting	Usual care/Usual care, no endurance training	VO2peak (Leg cycle ergometry): + (*p* < 0.01, ES = 0.90)
	Age: 69.4			Intensity: Resistance progression after 2 sets of 10 repetitions		10MWT: + (*p* = 0.05 < 0.10, ES = 0.70)
	Days since stroke (intervention group): 77.5 ± 28.7			Duration: 90 min		6MWT: + (*p* < 0.05, ES = 0.80)
	Moderate impaired, OPS: 3.4			Frequency: 3x/week (36 sessions)		Duration of bike exercise: + (*p* < 0.001)
						Berg Balance Scale: + (*p* < 0.01, ES = 1.07)
						FMMS lower extremity: 0
						FMMS upper extremity: 0
						Grip strength: 0
						Wolf Motor Function Test: 0
						Functional Reach test: 0
						Ankle isometric dorsiflexion: 0
						Ankle isometric extension: 0
Eich_2004	50 participants	First-time supratentorial stroke, stroke interval <6 weeks before study onset, able to walk a minimum 12 m with intermittent help or stand-by while walking, Barthel Index 50–80, Participation in a 12 week rehabilitation program, cardiovascular stable, according to 12-lead ECG, bicycle ergometry reaching >50 W and examination by a cardiologist, no other neurologic or orthopaedic disease impairing walking, able to understand the study content	none	Mode: 6 weeks of treadmill training, if necessary with body weight support (max 15% body weight)	Usual care/Usual care	10MWT: + (*p* < 0.001, ES = 1.15)
	Age: 63.2			Intensity: Training heart rate = (HRmax-HRrest)*0.6 + HRrest		6MWT: + (*p* < 0.002, ES = 1.07)
	Days since stroke (intervention group): 42.7 ± 15.4			Duration: 30 min		Rivermead Motor Assessment Score: 0
	Moderate impaired, Barthel Index: 66.7			Frequency: 5x/week (30 sessions)		Walking quality: 0
Katz-Leurer_2003A	92 participants	48 after stroke, clinical signs of first stroke	Brainstem lesions or bilateral signs, no lower-limb paralysis, >30 days after first event, pathological ECG during stress testing, significant change in blood pressure upon exertion, resting systolic blood pressure >200 mmHg, resting diastolic blood pressure > 100 mmHg, arrhythmia, heart failure, beta-blockers, suffering from inflammatory or degenerative joint diseases	Mode: 8 weeks of leg cycle ergometry	Usual care/Usual care	WRpeak: + (*p* < 0.01, ES = 2.54)
Katz-Leurer_2003B	Age: 63.5			Intensity: 60% of heart rate reserve		Resting heart rate: + (*p* = < 0.02)
Katz-Leurer_2007	Days since stroke (intervention group): >30 ± N/A			Duration: 30 min		Number of stairs climbed until fatigue: + (*p* < 0.01)
	Moderate impaired, SSS: 31.0			Frequency: 3x/week (24 sessions)		10MWT: 0
						Walking distance until fatigue: 0
						FIM: 0
						FAI at 6 month follow-up: 0
						Heart rate variability: 0
Letombe_2010	18 participants	Right or left hemiplegia following ischaemic or haemorrhagic hemispheric stroke, a full set of aetiological data (CT and/or MRI scans, Holter ECG, Doppler, cardiac ultrasound), a stable clinical state, well-balanced treatment (particular in terms of antihypertensives and anticoagulants)	Existence of disorders associated with hemiplegic motor damage, such as cognitive and memory disorders, hemisensory neglect, the existence of an intercurrent affection or unstable brain lesions	Mode: 4 weeks of leg cycle ergometry or treadmill or stepper	Usual care/ADL focussed usual care	VO2peak (Leg cycle ergometry): + (*p* < 0.05, ES = 1.43)
	Age: 60			Intensity: 70–80% of maximum power (W)		WRpeak (Leg cycle ergometry): + (*p* < 0.05)
	Days since stroke (intervention group): 21.0 ± 3.0			Duration: 40–60 min		Test duration (Leg cycle ergometry): + (*p* < 0.05)
	Moderate impaired, Barthel Index: 41			Frequency: 4x/week (16 sessions)		Barthel Index: + (*p* < 0.05)
						Katz-ADL Scale Score: + (*p* < 0.05)
Outermans_2010	43 participants	Clinical diagnosis of hemiplegia following first or recurrent stroke, time since most recent stroke and time of recruitment between 2–8 weeks, ability to walk 10 meters without assistance; FAC >3	Case of cardiovascular instability, acute impairments of the lower extremities influencing walking ability, sensory communicative disorders	Mode: 4 weeks of task-oriented circuit class training	Usual care/Usual care and low intensity PT	6MWT: + (*p* < 0.02, ES = 0.75)
	Age: 57			Intensity: 40–80% of maximum heart rate reserve		10MWT: + (*p* < 0.03, ES = 3.00)
	Days since stroke (intervention group): 22.5 ± 8.2			Duration: 45 min		Berg Balance Scale: 0
	Moderate impaired, FAC >3			Frequency: 3x/week (12 sessions)		Functional Reach Test: 0
Tang_2009	57 participants	Walk at least 5 meters independently, Chedoke-McMaster Stroke Assessment leg impairment score of 3–7 (where spasticity and weakness are marked)	Contraindications to maximal exercise testing, musculoskeletal impairments or pain that would limit pedalling ability	Mode: 2–4 weeks of leg cycle ergometry	Usual care/Usual care	VO2peak (Leg cycle ergometry): + (*p* = 0.004, ES = 1.05)
	Age: 65.2			Intensity: 50–75% of maximum heart rate reserve		WRpeak (Leg cycle ergometry): + (*p* < 0.001, ES = 0.93)
	Days since stroke (intervention group): 17.8 ± 3.1 (6–62)			Duration: 30 min		6MWT: + (*p* < 0.001, ES = 0.67)
	Moderately impaired, NIH: 4.7			Frequency: 3x/week (9 sessions)		Peak heart rate (Leg cycle ergometry): + (*p* = 0.002)
						Gait speed preferred: + (*p* < 0.001)
						Gait speed fast: + (*p* < 0.001)
						Stroke Impact Scale (SIS): + (*p* < 0.001)
Toledano-Zarhi_2011	28 participants	Minor ischemic stroke, 1–3 weeks post stroke	Blood pressure >200/110, unstable angina pectoris, arrhythmia, congestive heart failure, ST depression >2 mm during rest ECG, 3rd degree atrioventricular block with no pacemaker, severe peripheral vascular disease, orthopaedic or neurological disability, dementia or major depression, age >80 years	Mode: 6 weeks of leg cycle ergometry or treadmill or handbike	None/Home based exercise program for strength and flexibility	6MWT: + (*p* < 0.001, ES = 1.89)
	Age: 65.0			Intensity: 50–70% of maximum heart rate reserve)		4 Square Step test: + (*p* = 0.03)
	Days since stroke (intervention group): 11.0 ± 5.0			Duration: 35–55 min		Test duration (treadmill exercise): + (*p* < 0.001)
	Minor impaired, Modified Ranking Scale: <2			Frequency: 2x/week (12 sessions)		13 Stairs descending: 0
						13 Stairs ascending: 0
						Heart rate rest: 0
						Heart rate work: 0
						Blood pressure rest: 0
						Blood pressure work: 0

Abbreviations: NIH = National Institute of Health, CVA = Cerebrovascular accident, OPS = Orpington Prognostic Scale, FMM = Fugl-Meyer Motor Score, 10MWT = 10 Meter Walk Test, 6MWT = 6 Minute Walk Test, MOS36 = 36 Item Short Form Health Survey, SSS = Scandinavian Stroke Scale, FIM = Functional Independence Measure, FAI = Frenchay Activities Index, FAC = Functional Ambulatory Classification.

The studies included involved 423 participants with mild to moderate deficits in motor function and functional abilities. The main inclusion criteria for the participants in the studies were: stroke in the acute and sub-acute phase (0–6 month after initial event), ability to ambulate minimally (measured using Functional Ambulatory Classification (FAC), Orpington Prognostic Scale, Fugl-Meyer Score, Barthel-Index, 10 Meter Walk Test, Chedoke-McMaster Stroke Assessment), and appropriate cognitive function (measured using Mini Mental State Examination). The main exclusion criterion was serious cardiac contraindications for exercise testing according to the American College of Sports Medicine (ACSM) [43].

Leg cycle ergometry (5 studies) and treadmill training (4 studies) were the most common methods for aerobic exercise in the sub-acute phase following stroke. One study used task-orientated circuit class training [40]. Letombe et al. [39] and Toledano-Zarhi et al. [42] mixed the interventions using leg cycle ergometry or treadmill training or aerobic exercise with a stepper or hand bike [39,42]. Training duration ranged from 3 to 13 weeks (mean 6.56 ± 3.7 weeks), whereas training intensity ranged from 40–80% of heart rate reserve. All reviewed studies used an intervention protocol consisting of continuous exercise of 20–90 min (mean 47.78 ± 25.8 min) for 2–5 sessions per week (mean 3.44 ± 1.0 sessions/week). The intervention started 34.28 ± 25.1 days after the initial stroke event. The earliest time was 6 days post stroke [41,42].

Outcomes which were directly related to aerobic capacity included peak oxygen uptake (VO_2_peak), peak work rate (WRpeak), peak heart rate (HRpeak), heart rate variability, or the 6 Minute Walk Test (6MWT). Usually, VO_2_peak was evaluated using semi-recumbent leg cycle ergo-spirometry. Only Cunha et al. [32] measured oxygen uptake during a 5-minute walk using a portable gas analyser (KB1-C system). The 6MWT was used in 7 of 11 studies, whereas Cunha et al. [32] used a 5-minute walk test [32]. HRpeak was only evaluated by Tang et al. [41].

Further outcomes were gait speed, motor recovery and functional ability (Rivermead Motor Assessment Score, Functional Ambulatory Classification (FAC), Fugl-Meyer Scale, Barthel-Index, Lawton Instrumental ADL, Functional Independence Measure (FIM), Frenchay Activities Index (FAI)), Balance (Berg Balance Scale, Functional Reach Test), hand function (Wolf Motor Function Test, Jebsen Test, grip strength), stair ascent and descent, and quality-of-life using the Stroke Impact Scale (SIS).

### Risk of bias within studies

The methodological quality of the studies included is summarised in Table 2. The most common quality problems were absence of blinding of the assessors (8 studies), absence of concealment of allocation (7 studies), and absence of an intention-to-treat analysis (4 studies). The inter-rater agreement of the quality assessment was considered to be very good (Kappa: 0.81, SE: 0.06, CI95%: 0.70–0.92).

**Table 2:** Methodological quality of included studies (PEDro scale)

Criterion	Study ID										
	Cunha_ 2002	Duncan_ 1998	Duncan_ 2003	Eich_ 2004	Katz-Leurer_ 2003A	Katz-Leurer_ 2003B	Katz-Leurer_ 2007	Letombe_ 2010	Outermans_ 2010	Tang_ 2009	Toledano- Zarhi_2011
Eligibility criteria specified§	1	1	1	1	1	1	1	1	1	1	1
Random allocation to groups	1	1	1	1	1	1	1	1	1	1	1
Concealed allocation	0	1	1	1	0	0	0	0	1	0	0
Groups similar at baseline	0	1	1	1	1	1	1	1	1	1	1
Subject blinding	0	0	0	0	0	0	0	0	0	0	0
Therapist blinding	0	0	0	0	0	0	0	0	0	0	0
Assessor blinding	0	0	1	1	0	1	0	0	0	0	0
<15% dropouts	1	1	1	1	1	1	1	1	0	0	1
Intention-to-treat analysis	0	1	1	1	0	0	0	1	1	1	1
Between groups statistics reported	1	1	1	1	1	1	1	1	1	1	1
Point estimates and variability data reported	1	1	1	1	1	1	1	1	1	1	1
**Total score (0/10)**	**4**	**7**	**8**	**8**	**5**	**6**	**5**	**6**	**6**	**5**	**6**

Abbreviations: § Criterion does not contribute to total score.

### Syntheses of results

**Figure 2:**
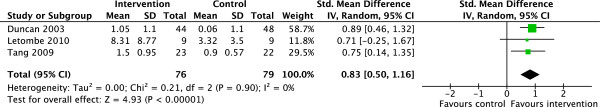
**Forest plot of 3 trials comparing the effects of additional cardiovascular exercise on aerobic capacity in sub-acute stroke.** Values are given in ml/kg/min peak oxygen uptake (VO_2_peak). Abbreviations: SD = standard deviation, IV = inverse variance, CI = confidence interval, df = degree of freedom.

**Figure 3:**
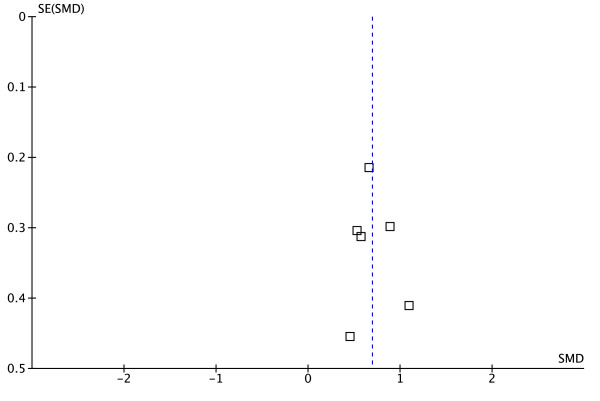
**Forest plot of 6 trials comparing the effects of additional cardiovascular exercise on walking endurance using the 6 Minute Walk Test (6MWT) in sub-acute stroke.** Values are given in maximal walking distance (m) within 6 minutes. Abbreviations: SD = standard deviation, IV = inverse variance, CI = confidence interval, df = degrees of freedom.

**Figure 4:**
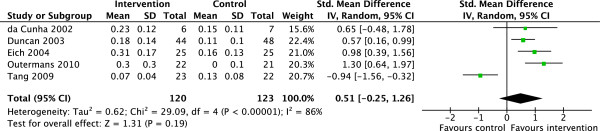
**Forest plot of 5 trials comparing the effects of additional cardiovascular exercise on gait speed using the 10 Meter Walk Test (10MWT) in sub-acute stroke.** Values are given in maximal gait speed (m/s) over 10 meters. Abbreviations: SD = standard deviation, IV = inverse variance, CI = confidence interval, df = degree of freedom.

The synthesis includes only outcomes VO_2_peak, 6MWT distance and gait speed, where there was sufficient evidence to pool the data. VO_2_peak (ml/kg/min) data were available in 3 studies, which randomized a total of 173 participants and reported data from 155 participants [34,39,41]. Pooled analysis for VO_2_peak yielded homogenous effects favouring the intervention group (SMD = 0.83, CI95% = 0.50–1.16, Z = 4.93, P < 0.01) (Figure 2). The 6MWT (m) was assessed in 6 studies involving 278 participants, while the baseline sample comprised 298 participants [33-35,40-42]. Pooled analysis for the results of the 6MWT revealed homogenous effects favouring the cardiovascular training intervention group (SMD = 0.69, CI95% = 0.45–0.94, Z = 5.58, P < 0.01) (Figure 3). Gait speed (m/s) measured in 5 studies involving 243 participants did not reveal significant effects attributable to a cardiovascular intervention (SMD = 0.51, CI95% = −0.25–1.26, Z = 1.31, P = 0.19) [32,34,35,40,41] (Figure 4).

### Risk of bias across studies

The syntheses of VO_2_peak (I^2^ = 0%, x^2^ = 0.21, df = 2, P = 0.90) and the 6MWT (I^2^ = 0%, x^2^ = 2.15, df = 5, P = 0.83) results were almost homogenous. We only detected significant heterogeneity for the outcome gait speed (I^2^ = 86%, x^2^ = 29.09, df = 4, P < 0.01). The funnel plots revealed low publication bias in all of the 3 syntheses (see Additional file 2: Figures S5, Additional file 3: Figure S6 and Additional file 4: Figure S7).

### Results of individual studies

Three studies reported between-group improvements in VO_2_peak after cardiovascular exercise (ES = 0.90, MD = 0.99, CI95% = 0.54–1.44) [34], (ES = 1.43, MD = 4.99, CI95% = −1.18–11.16) [39], (ES = 1.05, MD = 0.60, CI95% = 0.14–1.06) [41]. Katz-Leurer et al. [37] and Tang et al. [41] reported large effect sizes for WRpeak after cardiovascular exercise (ES = 2.54, MD = 11.00, CI95% = −117.00–193.00) (ES = 0.93, MD = 4.00, CI95% = 0.67–7.33), while Letombe et al. [39] did not publish baseline scores. Furthermore, Tang et al. reported a decrease in HRpeak after 2–4 weeks of cardiovascular exercise (ES = −0.96, MD = −7.40, CI95% = −10.86—3.94) compared to the control group [41]. In contrast, heart rate variability did not significantly decrease after cardiovascular exercise as reported by Katz-Leurer et al. [38]. The 6MWT values yielded between-group improvements in 6 studies after cardiovascular exercise intervention [33-35,40-42], whereas da Cunha et al. assessed maximal walking distance using a 5-minute walk-test, and reported no between-group differences after 3 weeks of treadmill exercise training (ES = 1.21, MD = 46.25, CI95% = 3.40–89.10) [32]. Studies that used walking as a major part of the intervention protocol showed larger effect sizes in the 6MWT (ES = 1.07, MD = 34.90, CI95% = 13.59–56.21) [35], (ES = 1.89, MD = 28.40, CI95% = 9.81–46.99) [42] than studies that did not implement walking training (ES = 0.67, MD = 81.00, CI95% = 68.00–230.00) [33], (ES = 0.80, MD = 28.02, CI95% = 10.76–45.28) [34], (ES = 0.67, MD = 37.70, CI95% = −2.85–78.25) [41].

Gait speed was increased in 4 of 5 studies after additional cardiovascular training [33-35,40]. Tang et al. reported comparable improvements for both groups [41]. Motor recovery and functional ability were improved in 2 of 7 outcome measurements across 4 studies [33,34,39]. Duncan et al. reported improvements for the Fugl-Meyer Upper Extremity Score (ES = 1.68, MD = 6.20, CI95% = −2.85–15.25) and the Fugl-Meyer Lower Extremity Score (ES = 21.0, MD = 5.67, CI95% = 4.76–6.58) [33]. Letombe et al. reported improvements in ADL associated scores such as the Barthel-Index (ES = 7.53, MD = 22.2, CI95% = 4.86–39.54), and the Katz-ADL Scale (ES = 1.16, MD = 2.90, CI95% = 1.39–4.41) [39] after cardiovascular exercise. Balance, measured in 3 studies, improved according to the Berg Balance Scale after 12 weeks of multimodal physical exercise training (ES = 1.07, MD = 2.66, CI95% = 1.43–3.89) [34]. A further 2 studies using the Berg Balance Scale or Functional Reach Test showed no improvements after physical exercise training [33,34,40]. Improved stair climbing and an associated decreased in fatigue was reported in one study by Katz-Leurer et al. [37]. In contrast to this is the finding of Toledano-Zarhi et al. who could not confirm these results based on measuring ascent and descent time of 13 steps [42]. Specific hand function assessed with the Wolf Motor Function Test [34] and the Jebsen Test [33] did not improve after 12 weeks of cardiovascular exercise. Grip strength showed no change after cardiovascular exercise in a study performed by Duncan et al. [34]. Finally, quality-of-life was measured and improved in 1 study using the Stroke Impact Scale (SIS) (ES = 0.24, MD = 3.30, CI95% = −5.38–11.98) [41].

### Adverse effects

Few adverse events were reported during or after cardiovascular exercise in individuals with sub-acute stroke during the early rehabilitation phase. Recurrent stroke occurred in 3 out of 50 participants (6%) in the intervention group during the cardiovascular exercise trial by Duncan et al.: 2 events occurred within 2 weeks after randomization and one event occurred at seven weeks. No adverse events were reported during the training sessions [34]. Katz-Leurer et al. reported two cases that were withdrawn from a study due to myocardial infarction and deep vein thrombophlebitis, and two recurrent stroke attacks during follow up in the control group [36,37]. Tang et al. reported exclusions due to acute low back pain, abnormal blood pressure responses, and abnormal electrocardiogram [41].

### Exercise testing protocol

Four of 11 studies assessed aerobic capacity by spiro-ergometry or work rate evaluation using leg cycle ergometry; 2 of these studies reported the use of semi-recumbent bikes [34,41], 1 study used wheelchair based ergometry [37], and 1 used standard leg cycle ergometry [39]. The protocols were similar in that resistance was increased between 5–10 W every 2 minutes and termination criteria defined according to ACSM were used [43]. However, there were large discrepancies regarding criteria for peak aerobic capacity. Duncan et al. [34] used a respiratory exchange ratio (RER) of >1.15 and 90% of age predicted HR maximum (Formula N/A), Katz-Leurer et al. [37] used 85% of age predicted HR maximum ((220-age)x0.85)), and Letombe et al. did not specify criteria for peak aerobic capacity [39]. Only Tang xet al. [41] provided uniquely defined criteria such as a plateau in VO_2_, RER >1.0, and peak HR within 10 beats per minute of age predicted heart rate maximum ((208-(0.7xage)) [41] as recommended by the ACSM guidelines for exercise testing and prescription [43]. There were no adverse effects reported during exercise testing in the studies reviewed.

## Discussion

This systematic review provides an overview of the currently available evidence for the use of cardiovascular training in the early stages after stroke. The aim was to identify strategies that have the potential to influence aerobic capacity, physical functioning, and quality-of-life.

The methodological quality of the trials included was good (7 studies) to fair (4 studies). Failure to conceal the sequencing of treatment allocation before subject recruitment and failure to blind the outcome assessor were the most prevalent methodological shortcomings of the papers included.

The results of the meta-analysis for the VO_2_peak outcome after cardiovascular exercise, in individuals starting exercise within 6 days to 6 months after stroke onset, revealed a large effect size (SMD = 0.83). This finding supports the evidence that individuals in the sub-acute stage after stroke have high potential to increase VO_2_peak following a cardiovascular training intervention. This is in addition to a spontaneous recovery of peak aerobic capacity of 16.9% that occurs during the first 6 months after stroke, as shown by MacKay-Lyons & Makrides [44]. The observed additional mean improvement in peak oxygen uptake (+0.81 ml/kg/min) after additional cardiovascular training in our meta-analysis seems to be low. But given the fact that VO_2_peak is reduced to 10–17 ml/kg/min within 0–30 days after stroke [12-14], and 10 ml/kg/min is required for light instrumental activities during all activities of daily living (ADL) [45], this small improvement of peak aerobic capacity could lead to a large functional carryover. Furthermore, we have to consider that individuals with stroke need a higher aerobic capacity for basic ADL functions such as walking due to their impairments [46]. These facts confirm the importance of increasing aerobic capacity as much as possible. However, the short intervention lengths (mean 6.56 ± 3.7 weeks) that were used in the included trials may be indicative of the fact that only small training effects were to be expected.

WRpeak, which is strongly related to VO_2_peak, revealed large effect sizes in 2 studies [37,41]. However, these results may be strongly affected by the fact that leg cycle ergometry was used both as training intervention and test measure. Therefore, the observed effect might be partly attributable to learning effects and improved skill due to cycle practice.

Consistent medium ES was found for walking distance within 6 minutes measured by the 6MWT. Detailed analysis revealed that individuals in the intervention group walked 31.68 m (CI95%: 21.95–41.40) farther than participants without additional cardiovascular training. 3 of 6 studies either used treadmill exercise [35,42] or focussed on walking competence [40] to improve cardiovascular fitness. Therefore, the latter study might also affect the 6MWT results by the additional gait practice that was performed. This might explain the lower ES in trials using leg cycle ergometry as the intervention method compared to trials using walking exercise. However, these significant improvements in walking distance after aerobic exercise using either a treadmill or walking based exercises demonstrate a functional carryover of enhanced aerobic capacity. Neither form of intervention was able to induce a change large enough to reach a minimal clinically important difference of 54.1 m during a 6 minute walk [47]. Whether this is due to insufficient training intensity, low training frequency, non-use of progression schemes, or other training related factors cannot be answered here. This should be a focal point of future trials where different training related parameters are compared; e.g. intensities, frequencies, and progression schemes.

Despite the fact that 4 out of 5 studies revealed moderate to large ES for improving gait speed, it remains unclear whether additional cardiovascular exercise can affect this outcome. The intervention group showed only a 0.1 m/s faster gait speed compared to the control group, whereas 0.3 m/s may be a minimal clinically relevant difference, as reported in a comparable sample of individuals with sub-acute stroke [47].

The outcomes motor recovery, functional ability and balance did not consistently improve after additional cardiovascular exercise. We found large heterogeneity in the outcome measurements that were used and, therefore, we were not able to synthesise these results. An improvement in motor recovery and functional ability after prolonged aerobic training, combined with the implementation of more sensitive measures, may lead to some evidence concerning the effects of cardiovascular exercise on physical function.

When the focus is put on functional activities, stair negotiation was reported in several studies. It remains unclear how the level of aerobic capacity influences the ability to ascend and descend stairs in sub-acute stroke. Katz-Leurer et al. have shown improvements in stair climbing until fatigue. However, this study failed to report baseline data for this parameter by only stating percentage improvements. In contrast, Toledano-Zarhi et al. could not report improvements in stair negotiation by measuring the time needed to negotiate 13 stairs while ascending and descending. Stair climbing depends on strength, balance and aerobic capacity, and is a rather complex motor task. What can be said is that if we are looking at aerobic capacity, stair-walking tests should have a longer duration than climbing 1 flight of stairs and reach symptom limited exhaustion of the participant.

In contrast to the findings of Ploughman et al. [48] derived from individuals with chronic stroke, upper-extremity tasks did not improve following additional cardiovascular exercise. Recent studies have shown that movement speed of the upper-extremities was increased after physical exercise training, probably due to an increase in core temperature and neuronal transmission [49]. A potentially biasing effect in this context could be that the tests were performed directly after the exercise, which could have influenced the test results. We therefore conclude that it is unclear whether cardiovascular exercise after stroke also has an impact on upper-extremity motor function.

Quality-of-life, evaluated by Tang et al. using the Stroke Impact Scale (SIS), revealed only low ES towards additional cardiovascular exercise [41]. This result might be explained by the short intervention length of 2–4 weeks. There were no other reports on this parameter from the other studies. However, the association of cardiovascular fitness, physical functioning and quality-of-life may be explored in further investigations, regarding the importance of carry over baseline effects into all ADL tasks.

Various exercise protocols have been used to improve cardiovascular fitness. Three studies used mixed training interventions [33,34,40] (strength, balance, endurance), and 2 studies did not explicitly exclude endurance training in the control group [32,39,42]. This inconsistent use of exercise protocols might lead to potential intervention bias regarding the evidence of optimal training protocols to be used in sub-acute stroke.

### Risk of cardiovascular exercise early after stroke

Regarding the safety of early cardiovascular exercise after stroke, the incidence rate of adverse effects such as recurrent stroke reported in the analysed trials was comparable to the usual recurrent stroke risk, which is 8% between one and six months after stroke [50]. Further analysis based on the studies included yields a relative risk ratio of 1.25 for cardiovascular exercise compared to standard care following sub-acute stroke rehabilitation. Given the fact that cardiovascular training intervention started between 6 to 76 days (mean 34.28 ± 25.1 days) after stroke onset, and there is a low relative risk for adverse effects, it can be said that cardiovascular exercise early after stroke is safe. However, there were strict exclusion criteria in all studies, mostly based on the ACSM guidelines [43] (Table 1), thus individuals with stroke showing severe cardiac disease were not considered. Moreover, the applicability of cardiovascular exercise in the acute period after stroke (<7 days post event) remains unclear.

### Exercise testing early after stroke

All exercise testing protocols used in the studies reviewed are consistent with the ACSM guidelines for exercise testing and prescription [43]. However, there is a lack of a precise description regarding termination criteria for exercise testing and evaluation of aerobic capacity (e.g. criteria for reaching VO_2_peak) in most of the included studies. Currently, leg cycle ergometry is the most appropriate and valid assessment method to measure aerobic capacity in individuals with lower fitness levels. None of the reviewed studies reported major adverse events that were directly attributable to the cardiovascular training. This can be regarded as an encouraging finding since it raises the possibility to start exercise training and testing in the very early period after stroke. It is important to note that participants tested in these studies were only moderately to mildly affected and certainly able to perform the established exercise testing methods such as semi-recumbent leg cycle ergometry. Concepts to evaluate aerobic capacity in severely affected individuals after stroke are lacking.

### Limitations

We developed and utilized a structured study protocol to guide our search strategy, study selection, extraction of data and statistical analysis. Limitations of this review should however be noted. First, a publication bias may have been present, as well as a language bias, given that we considered only interventions described in published studies and restricted our search to English, French, and German language publications. Second, as there were few randomized trials, we also included one non-randomized study, the results of which may be affected by confounding bias due to the absence of random assignment. Third, as noted previously, participants included were moderately to mildly affected and, therefore, the results of the study are not fully generalizable to the stroke population at large. Furthermore, this analysis cannot provide statements concerning cardiovascular training effects in the very early period after stroke (<7 days post event). Fourth, reports of specific medication usage of the participants were not given in most studies; beta-blockers, for example, may affect cardiovascular responses during exercise [51]. Fifth, the relatively short duration of the interventions without follow-up for the course of outcomes after the cardiovascular exercise programme might be considered a limitation. Sixth, the quality of the studies varied. Concealed allocation and the blinding of the assessors were not explicitly stated in the methodology or were absent. Seventh, the analyses of aerobic capacity strongly varied between studies or were not described sufficiently to ensure valid comparisons. Finally, publication bias was low but might account for some effects in the quantitative analysis.

### Clinical relevance

Regarding the results of the present meta-analysis, cardiovascular exercise interventions and exercise testing protocols using leg cycle ergometry have been found to be safe and feasible in the sub-acute stage after stroke. There is robust evidence that individuals with sub-acute stroke may benefit from these protocols to improve peak oxygen uptake and walking distance. Therefore, cardiovascular exercise protocols should be implemented into sub-acute stroke rehabilitation since conventional physical therapy programmes between 2 and 14 weeks post stroke did not elicit adequate cardiovascular stress to induce a training effect [13]. Clinicians and researchers should follow ACSM guidelines for exercise testing and prescription to ensure medical safety of training protocols and comparability for future analyses.

### Future directions

Leg cycle ergometry or treadmill exercise has been shown to be adequate for moderately to mildly affected individuals with stroke, but these concepts are not applicable in severely affected individuals. Moreover, methods to assess aerobic capacity in the early post-stroke phase and in severely affected individuals with stroke are not available. Future research must develop new concepts for assessment of aerobic capacity in severely disabled individuals and for the early post-stroke period. It is well established that repetitions play a major role in structural re-organisation of the brain [52], and task-orientated activities are a key to functional recovery [53]. Therefore, cardiovascular exercise methods as well as exercise testing protocols must consider ADL-related functions such as walking or stair climbing as a means to influence and assess aerobic performance early after stroke. It is a future challenge to develop appropriate devices that operate with assistance-as-needed to challenge individuals optimally, by taking the quality of the individual’s movements into account. Rehabilitation robotics could potentially support new directions in cardiovascular exercise testing and training early after stroke.

The effects of cardiovascular exercise on neuronal recovery, e.g. neuroplasticity, recovery of function, cognition, etc., are still unknown. Future studies must address these gaps by designing study protocols using advanced assessments to evaluate the effects of cardiovascular exercise after stroke. Potential evidence in this field may justify the application of cardiovascular exercise protocols even for severely affected individuals.

The analysis of aerobic capacity is challenging since there are no standardised evaluation methods [54]. Future research should provide a consistent analysis process, even for individuals after stroke.

## Conclusion

This systematic review shows robust evidence that cardiovascular exercise early after stroke enhances aerobic capacity by improving VO_2_peak and walking distance during 6 minutes (6MWT) in moderately to mildly affected individuals. These findings have the potential for implementation in sub-acute stroke rehabilitation. Concepts to influence and evaluate aerobic capacity in severely affected individuals with sub-acute stroke, as well as in the very early period after stroke (<7 days post stroke), are lacking. Further research is needed to develop appropriate methods for cardiovascular rehabilitation early after stroke, and to evaluate long-term effects of cardiovascular exercise on aerobic capacity, physical functioning, and quality-of-life.

## Competing interests

The authors declare that they have no competing interests.

## Authors’ contributions

OS: Methodology, Data collection, Quality assessments, Analysis, Manuscript writing. EDB: Methodology, Data collection, Quality assessments, Supervision, Manuscript writing & revision. RK: Analysis, Manuscript revision. KH: Study initiation, Supervision, Manuscript revision. All authors read and approved the final manuscript.

## Pre-publication history

The pre-publication history for this paper can be accessed here:

http://www.biomedcentral.com/1471-2377/12/45/prepub

## Supplementary Material

Additional file 1 Review protocol. Click here for file

Additional file 2 **Figure S5.** Funnel plot of 3 trials comparing the effects of additional cardiovascular exercise on aerobic capacity in sub-acute stroke. Abbreviations: SE = standard error of SMD, SMD = standardised mean difference. Click here for file

Additional file 3 **Figure S6.** Funnel plot of 6 trials comparing the effects of additional cardiovascular exercise on walking endurance using the 6 Minutes Walk Test (6MWT) in sub-acute stroke. Abbreviations: SE = standard error of SMD, SMD = standardised mean difference. Click here for file

Additional file 4 **Figure S7.** Funnel plot of 5 trials comparing the effects of additional cardiovascular exercise on gait speed using the 10 Meter Walk Test (10MWT) in sub-acute stroke. Values are given in maximal gait speed (m/s) over 10 meters. Abbreviations: SE = standard error of SMD, SMD = standardised mean difference. Click here for file
