# Supplementary material for: Effects of cardiovascular exercise early after stroke: systematic review and meta-analysis
Source: BMC Neurol. 2012 Jun 22;12:45. doi: 10.1186/1471-2377-12-45 (PMC3495034; doi:10.1186/1471-2377-12-45)
Supplement: Additional file 1 — Review protocol. [file 1471-2377-12-45-S1.pdf]

Additional file 1

Review protocol

Cardiovascular rehabilitation early after stroke – a systematic review & meta-analysis

Stoller O[1,2], de Bruin ED[3§,] Knols RH[4], Hunt KJ[1]

[1] Institute for Rehabilitation and Performance Technology, Bern University of Applied Sciences, Burgdorf, Switzerland

[2] Department of Epidemiology, Maastricht University and Caphri Research School, Maastricht, Netherlands

[3] Institute of Human Movement Sciences and Sport, ETH Zurich, Zurich, Switzerland

[4] Department of Rheumatology and Institute of Physical Medicine, University Hospital Zurich, Zurich, Switzerland

[§]Corresponding author

Email addresses:

OS oliver.stoller@bfh.ch

EDB debruin@move.biol.ethz.ch

RK ruud.knols@usz.ch

KH kenneth.hunt@bfh.ch

The context of the review and its conceptual issues

Stroke is the leading cause of disability in adults and raise cost about €22 in the European Union [1, 2]. Since today, stroke rehabilitation strategies have focused primarily on improving the capacity of the neuromuscular system [3, 4]. Nevertheless, around 75% of post stroke have cardiac disease, and long-term stroke survivors are at greater risk of dying from cardiac disease than from any other cause, including a second stroke [5, 6]. It has also been suggested that stroke patients are more disabled by associated cardiac disease than by the stroke itself [7]. Pre-existing or post stroke cardiovascular conditions can delay or inhibit participation in a therapeutic exercise program, complicate the rehabilitation and long-term post stroke course of care, and limit the ability of the patient to perform functional activities independently [6, 8]. Therefore, cardiovascular training should be a major part in stroke rehabilitation and it is well documented in chronic stroke survivors [9-11]. There is evidence that cardiovascular fitness can be improved via cardiovascular training and that there is an association to reduced dependence during ambulation and improved walking performance in terms of speed and walking distance [11]. However, less is known about the effects in the early stages after stroke and about the long-term effects on physical functioning. Cardiovascular training seems to significantly improve VO2 peak and exercise tolerance in early stages after a stroke [12-15], whereas the impact on functional benefits is less clear. In addition, the "Physical Activity and Exercise Recommendations for Stroke Survivors" guidelines concluded that there are no specifically studies who address the issue of how soon after stroke graded exercise can be performed safely and which intensities should be used [16]. First studies in the recent years confirmed feasibility of cardiopulmonary exercise testing in subjects early after stroke (~10days post stroke) [17, 18].

What is the health care / treatment context?

Given the fact that most motor and functional recovery occurs in the first 3 month after stroke [19], there could be a hitherto neglected potential to precipitate motor recovery by improving cardiovascular capacity in acute rehabilitation after stroke, which could lead to improved physical functioning and quality of life.

What is the aim of the systematic review?

This systematic review will look at the short- and long-term effects of cardiovascular training in acute stages after stroke. We will outline the current evidence of early cardiovascular activation after stroke. We agree that our aim is to end in a peer-reviewed publication.

What is the research question?

Does cardiovascular training early after stroke improve aerobic capacity, physical function and quality of life?

What is the search strategy?

We search the databases MEDLINE, EMBASE, Cochrane Library, CINAHL, and ISI Web of Science (WOS). We use medical sub-headings as search terms, including the following main terms for the population: Stroke; Cerebral Stroke; Vascular Accident; Brain Vascular Accident; Apoplexy; Cerebrovascular Apoplexy; Cerebrovascular Stroke; CVA (Cerebrovascular Accident); Cerebrovascular Accident; Acute Stroke; Acute Cerebrovascular Accident; Acute 0-6 month post stroke; age >18 years; for the intervention of interest: cardiovascular training; cardiopulmonary training; cardiorespiratory training; aerobic training; endurance training; exercise; endurance exercise; ergometry; cycling; rowing; treadmill, and for the outcomes of interest: cardiovascular fitness; aerobic fitness, condition, endurance, physical conditioning; VO2 maximal; VO2 maximum; VO2 peak; maximal oxygen uptake; heart rate; neural recovery; neural rehabilitation; functional recovery; function recovery; quality of life. We perform additional hand search by scanning reference lists of identified studies.

What are the inclusion/exclusion criteria?

We include only randomised and nonrandomized prospective controlled cohort studies considering cardiovascular training early after stroke, including patients of any age in the acute phase that were medically stable enough when starting the interventions (<6 month after stroke, all types of stroke, all types of severity levels). Furthermore, we include only studies using cardiovascular training

interventions. Typically, it should be performed for extended periods of time on devices or ergometers (e.g. treadmill, cycling, rowing), or by utilising modes of activity such as walking or stair climbing. We include any types of objective and subjective measures.

How will the data be extracted and analysed?

Two reviewers (OS/EDB) independently extract data from the selected studies. To facilitate comparison between studies, we will extract data based on quality indicators from the CONSORT statement [20]. To ensure the clarity and transparency of reporting of the systematic review we will follow the PRISMA guidelines [21]. We use Review Manager [22] and Microsoft Excel (2011) for analysis. Missing values will be calculated according to the Cochrane Handbook for Systematic Reviews [23].

How will the quality of studies be assessed?

Two reviewers (OS/EDB) independently assess the methodological quality of the studies according to the PEDro scale [24]. Percentage agreement and Cohen's Kappa statistic [25] are calculated interpreted in accordance with Landis and Koch's benchmarks for assessing the agreement between raters: poor (<0), slight (.0-.20), fair (.21-.40), moderate (.41-.60), substantial (.61-.80), and almost perfect (.81-1.0) [26].

How is the time schedule?

**Time**	**Tasks**	**Responsibility**
Januar – March 2011	Protocol definition	OS
April 2011 / November 2011	Search	OS & M. Gosteli
May – August 2011	Analysis	OS
September 2011	Paper draft I	OS
Oktober 2011	Revision I	OS, EDB, RK, KH
November 2011	Revision II	OS, EDB, RK, KH
December 2011	Final approval	OS, EDB, RK, KH

References

1. Sarti C, Rastenyte D, Cepaitis Z, Tuomilehto J: **International trends in mortality from stroke, 1968 to 1994**. *Stroke* 2000, **31**(7):1588-1588.
2. Truelsen T, Piechowski-Jó017Awiak B, Bonita R, Mathers C, Bogousslavsky J, Boysen G: **Stroke incidence and prevalence in Europe: a review of available data**. *European Journal of Neurology* 2006, **13**(6):581-598.
3. Carr JH, Mungovan SF, Shepherd RB, Dean CM, Nordholm LA: **Physiotherapy in stroke rehabilitation: bases for Australian physiotherapists' choice of treatment**. *Physiotherapy Theory and Practice* 1994, **10**(4):201-209.
4. Wade D: **Rehabilitation therapy after stroke**. *The Lancet* 1999, **354**(9174):176-177.
5. Roth EJ: **Heart disease in patients with stroke: incidence, impact, and implications for rehabilitation. Part 1: Classification and prevalence**. *Archives of physical medicine and rehabilitation* 1993, **74**(7):752-752.
6. Roth EJ: **Heart disease in patients with stroke. Part II: Impact and implications for rehabilitation**. *Archives of physical medicine and rehabilitation* 1994, **75**(1):94-94.
7. Gresham GE, Phillips TF, Wolf PA, McNamara PM, Kannel WB, Dawber TR: **EPIDEMIOLOGIC PROFILE OF LONG-TERM STROKE DISABILITY - FRAMINGHAM-STUDY**. *Archives of Physical Medicine and Rehabilitation* 1979, **60**(11):487-491.
8. Roth EJ, Mueller K, Green D: **STROKE REHABILITATION OUTCOME - IMPACT OF CORONARY-ARTERY DISEASE**. *Stroke* 1988, **19**(1):42-47.
9. Pang MYC, Eng JJ, Dawson AS, Gylfadóttir S: **The use of aerobic exercise training in improving aerobic capacity in individuals with stroke: a meta-analysis**. *Clinical Rehabilitation* 2006, **20**(2):97-111.
10. Rimmer JH, Wang E: **Aerobic exercise training in stroke survivors**. *Topics in Stroke Rehabilitation* 2005, **12**(1):17-30.

11. Saunders DH, Greig CA, Mead GE, Young A: **Physical fitness training for stroke patients**. *Cochrane Database of Systematic Reviews* 2009(4).

12. da Cunha IT, Lim PA, Qureshy H, Henson H, Monga T, Protas EJ: **Gait outcomes after acute stroke rehabilitation with supported treadmill ambulation training: A randomized controlled pilot study**. *Archives of Physical Medicine and Rehabilitation* 2002, **83**(9):1258-1265.

13. Duncan P, Richards L, Wallace D, Stoker-Yates J, Pohl PP, Luchies C, Ogle A, Studenski S: **A randomized, controlled pilot study of a home-based exercise program for individuals with mild and moderate stroke**. *Stroke* 1998, **29**(10):2055-2060.

14. Potempa K, Lopez M, Braun LT, Szidon JP, Fogg L, Tincknell T: **Physiological outcomes of aerobic exercise training in hemiparetic stroke patients**. *Stroke* 1995, **26**(1):101-101.

15. Katz-Leurer M, Shochina M, Carmeli E, Friedlander Y: **The influence of early aerobic training on the functional capacity in patients with cerebrovascular accident at the subacute stage**. *Archives of Physical Medicine and Rehabilitation* 2003, **84**(11):1609-1614.

16. Gordon NF, Gulanick M, Costa F, Fletcher G, Franklin BA, Roth EJ, Shephard T: **Physical activity and exercise recommendations for stroke survivors - An American Heart Association scientific statement from the Council on Clinical Cardiology, Subcommittee on Exercise, Cardiac Rehabilitation, and Prevention; the Council on Cardiovascular Nursing; the Council on Nutrition, Physical Activity, and Metabolism; and the Stroke Council**. *Stroke* 2004, **35**(5):1230-1240.

17. Chen JK, Chen TW, Chen CH, Huang MH: **PRELIMINARY STUDY OF EXERCISE CAPACITY IN POST-ACUTE STROKE SURVIVORS**. *Kaohsiung Journal of Medical Sciences* 2010, **26**(4):175-180.

18. Tang A, Sibley KM, Thomas SG, McIlroy WE, Brooks D: **Maximal exercise test results in subacute stroke**. *Archives of Physical Medicine and Rehabilitation* 2006, **87**(8):1100-1105.

19. Jorgensen HS, Nakayama H, Raaschou HO, Vivelarsen J, Stoier M, Olsen TS: **OUTCOME AND TIME-COURSE OF RECOVERY IN STROKE .2. TIME-COURSE OF**

RECOVERY - THE COPENHAGEN STROKE STUDY. *Archives of Physical Medicine and Rehabilitation* 1995, **76**(5):406-412.

20. Altman DG, Schulz KF, Moher D, Egger M, Davidoff F, Elbourne D, Gotzsche PC, Lang T, Grp C: **The revised CONSORT statement for reporting randomized trials: Explanation and elaboration**. *Annals of Internal Medicine* 2001, **134**(8):663-694.
21. Liberati A, Altman DG, Tetzlaff J, Mulrow C, Gotzsche PC, Ioannidis JP, Clarke M, Devereaux PJ, Kleijnen J, Moher D: **The PRISMA statement for reporting systematic reviews and meta-analyses of studies that evaluate healthcare interventions: explanation and elaboration**. *BMJ* 2009, **339**:b2700.
22. **Review Manager (RevMan) [Computer program]. Version 5.0. Copenhagen: The Nordic Cochrane Centre, The Cochrane Collaboration, 2008.** In.
23. Higgins J, Green S, (Editors): ***Cochrane Handbook for Systematic Reviews of Interventions*** **Version 5.1.0 [updated March 2011]. The Cochrane Collaboration, 2011. Available from** http://www.cochrane-handbook.org**.** In.; 2008.
24. Verhagen AP, de Vet HCW, de Bie RA, Kessels AGH, Boers M, Bouter LM, Knipschild PG: **The delphi list: A criteria list for quality assessment of randomized clinical trials for conducting systematic reviews developed by Delphi consensus**. *Journal of Clinical Epidemiology* 1998, **51**(12):1235-1241.
25. Cohen A: **COMPARISON OF CORRELATED CORRELATIONS**. *Statistics in Medicine* 1989, **8**(12):1485-1495.
26. Landis JR, Koch GG: **MEASUREMENT OF OBSERVER AGREEMENT FOR CATEGORICAL DATA**. *Biometrics* 1977, **33**(1):159-174.
