# Supplementary figures and images for: Effects of cardiovascular exercise early after stroke: systematic review and meta-analysis
Source: BMC Neurol. 2012 Jun 22;12:45. doi: 10.1186/1471-2377-12-45 (PMC3495034; doi:10.1186/1471-2377-12-45)

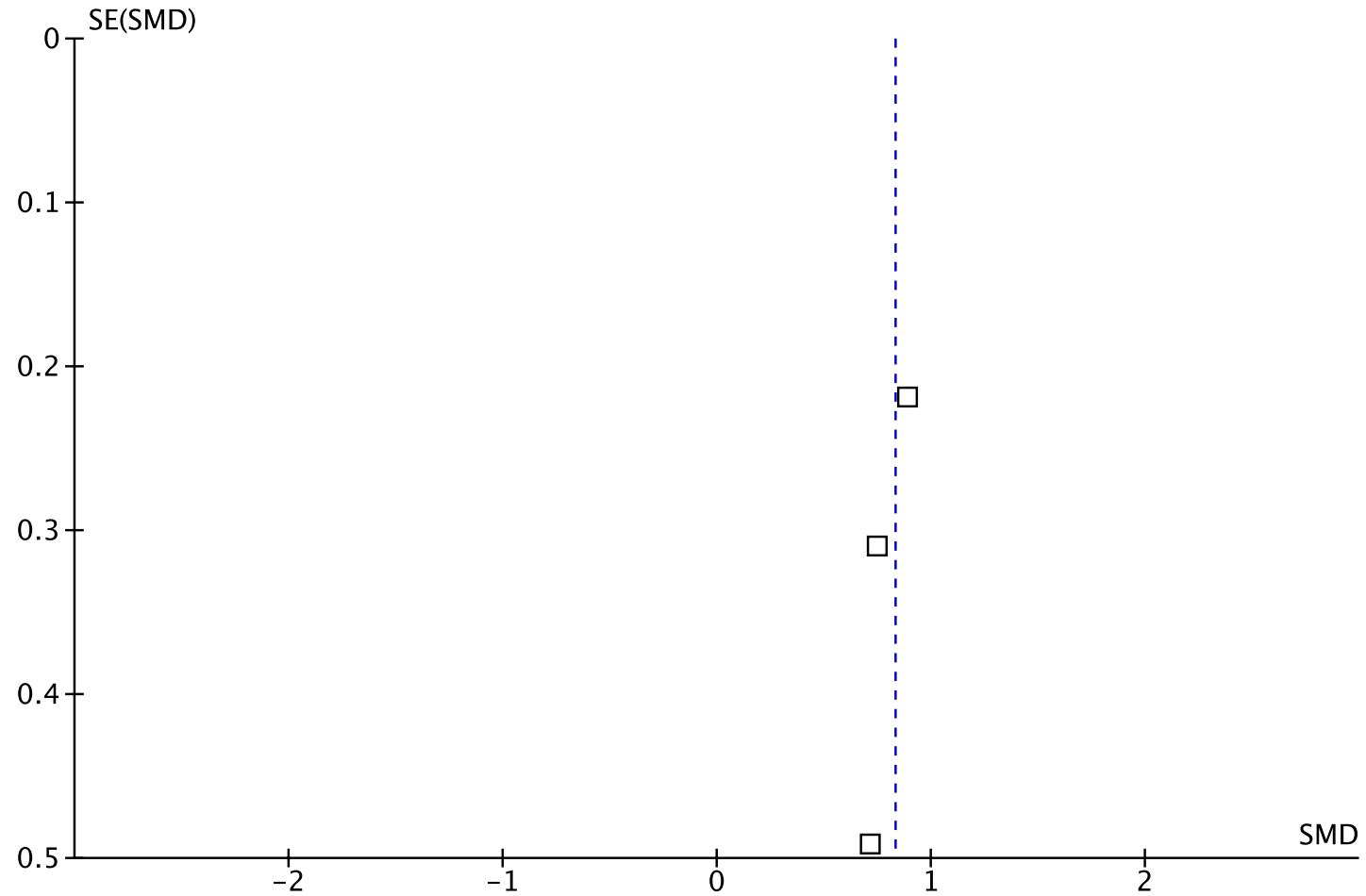

Supplement: Additional file 2 — Figure S5. Funnel plot of 3 trials comparing the effects of additional cardiovascular exercise on aerobic capacity in sub-acute stroke. Abbreviations: SE = standard error of SMD, SMD = standardised mean difference. [file 1471-2377-12-45-S2.pdf]

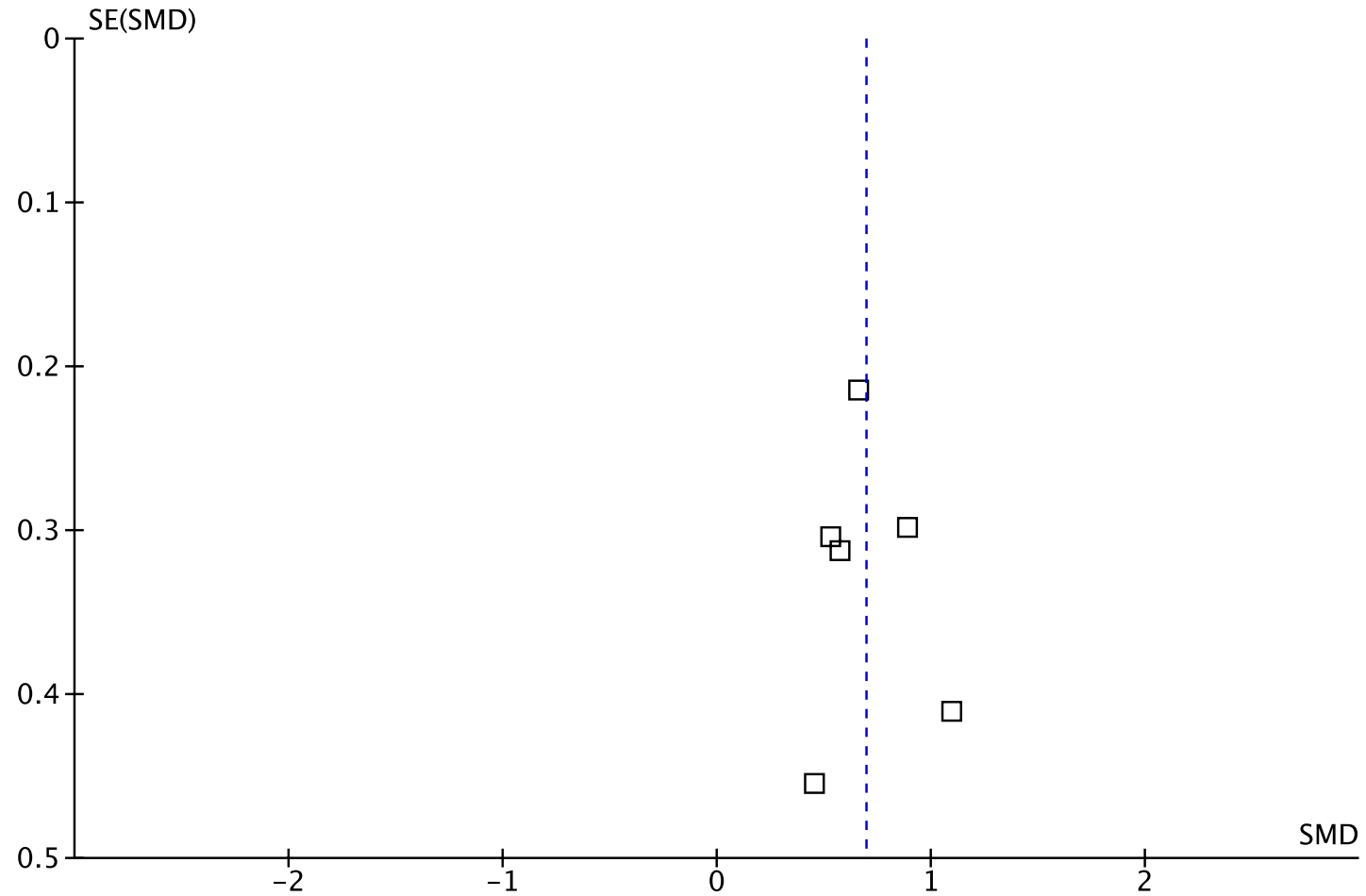

Supplement: Additional file 3 — Figure S6. Funnel plot of 6 trials comparing the effects of additional cardiovascular exercise on walking endurance using the 6 Minutes Walk Test (6MWT) in sub-acute stroke. Abbreviations: SE = standard error of SMD, SMD = standardised mean difference. [file 1471-2377-12-45-S3.pdf]

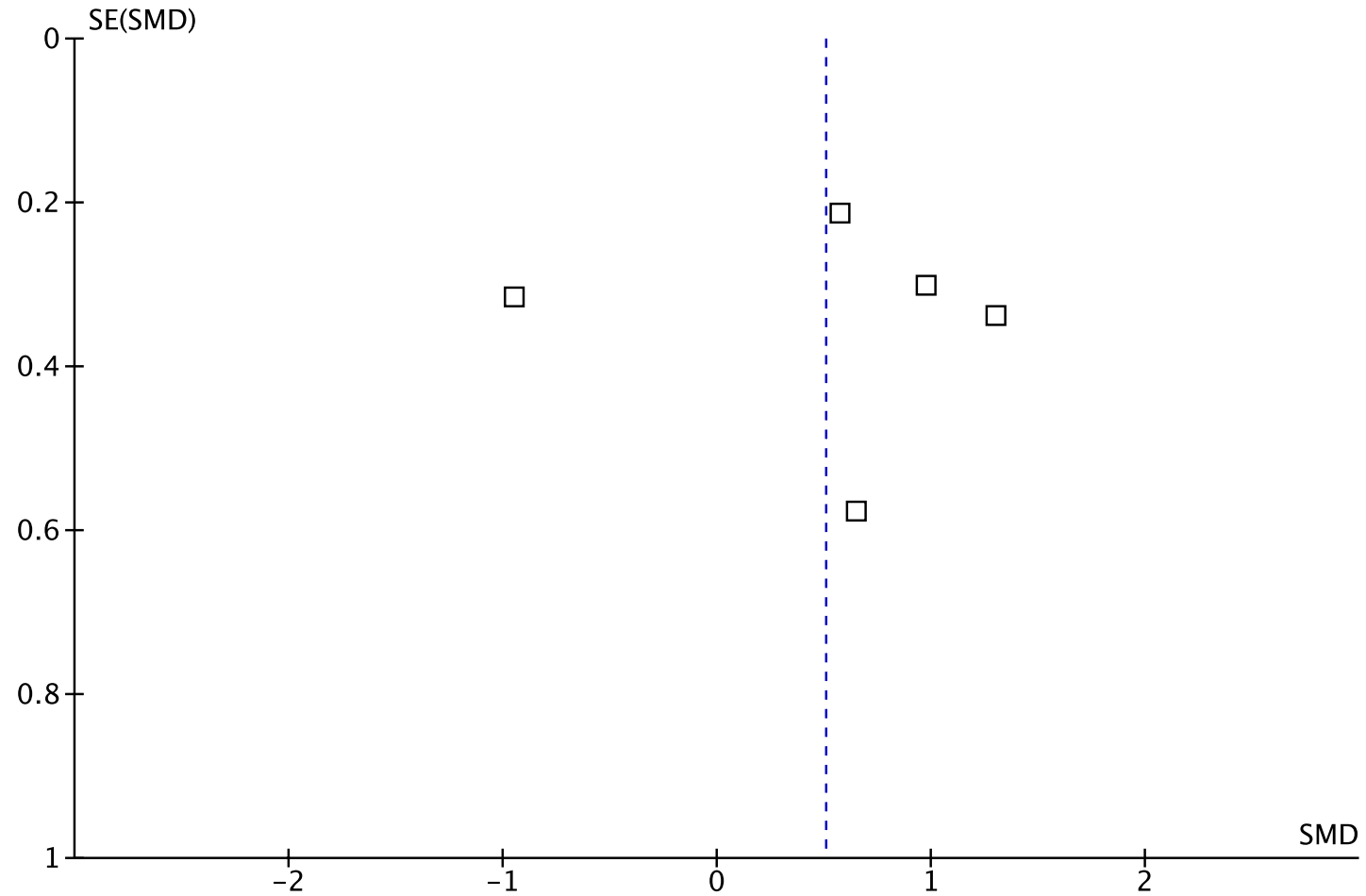

Supplement: Additional file 4 — Figure S7. Funnel plot of 5 trials comparing the effects of additional cardiovascular exercise on gait speed using the 10 Meter Walk Test (10MWT) in sub-acute stroke. Values are given in maximal gait speed (m/s) over 10 meters. Abbreviations: SE = standard error of SMD, SMD = standardised mean difference. [file 1471-2377-12-45-S4.pdf]
